# Antibacterial Immune Competence of Honey Bees (*Apis mellifera*) Is Adapted to Different Life Stages and Environmental Risks

**DOI:** 10.1371/journal.pone.0066415

**Published:** 2013-06-17

**Authors:** Heike Gätschenberger, Klara Azzami, Jürgen Tautz, Hildburg Beier

**Affiliations:** BEEgroup, Biocentre, University of Würzburg, Würzburg, Germany; Université Libre de Bruxelles, Belgium

## Abstract

The development of all honey bee castes proceeds through three different life stages all of which encounter microbial infections to a various extent. We have examined the immune strength of honey bees across all developmental stages with emphasis on the temporal expression of cellular and humoral immune responses upon artificial challenge with viable *Escherichia coli* bacteria. We employed a broad array of methods to investigate defence strategies of infected individuals: (a) fate of bacteria in the haemocoel; (b) nodule formation and (c) induction of antimicrobial peptides (AMPs). Newly emerged adult worker bees and drones were able to activate efficiently all examined immune reactions. The number of viable bacteria circulating in the haemocoel of infected bees declined rapidly by more than two orders of magnitude within the first 4–6 h post-injection (p.i.), coinciding with the occurrence of melanised nodules. Antimicrobial activity, on the other hand, became detectable only after the initial bacterial clearance. These two temporal patterns of defence reactions very likely represent the constitutive cellular and the induced humoral immune response. A unique feature of honey bees is that a fraction of worker bees survives the winter season in a cluster mostly engaged in thermoregulation. We show here that the overall immune strength of winter bees matches that of young summer bees although nodulation reactions are not initiated at all. As expected, high doses of injected viable *E.coli* bacteria caused no mortality in larvae or adults of each age. However, drone and worker pupae succumbed to challenge with *E.coli* even at low doses, accompanied by a premature darkening of the pupal body. In contrast to larvae and adults, we observed no fast clearance of viable bacteria and no induction of AMPs but a rapid proliferation of *E.coli* bacteria in the haemocoel of bee pupae ultimately leading to their death.

## Introduction

As eusocial insects, honey bees (*Apis mellifera*) maintain three castes within a colony. In temperate climate regions, a single queen (fertile female), up to 50,000 workers (sterile females) and about 2,000 drones (males) are found in hives during the early summer season [1]. Workers, queens and drones differ markedly in morphological, physiological and behavioural features. A characteristic property of honey bees is their specific way to survive the winter season as compared to other eusocial insects. The bulk of workers does not die in the autumn (like bumble bees) or hibernate in caves below the frost line (like ants and termites), but about 1/5 to 1/10 of the members of a bee colony cluster together when the temperatures drop. Thermal control of the winter cluster is achieved by both the insulation of tightly packed mantle bees and endothermic heat production of inner bees generated by shivering of their flight muscles [2]. Summer and winter bees differ in many ways, markedly in longevity. Summer bees live 20 to 40 days, whereas winter bees remain alive up to 230 days [3].

All three castes of the holometabolic honey bees proceed through three different developmental stages after hatching from the egg: larvae, pupae and adults. Larvae of all three castes leave the egg after a three-day long embryonic period but thereafter the developmental paths for workers, drones and queens are quite different. The larval phase is characterized by a period of constant feeding by nurse bees. During the early phase of development, larvae of both female castes are fed with royal jelly, a secretion produced by glands in the head of adult workers. At later larval stages, royal jelly is kept as the only diet for queens whereas a mixture of glandular secretions together with honey and pollen are supplied to worker larvae. The differential feeding of genetically related larvae regulates caste differentiation [4]. The molecular determinants of this phenomenon include multiple components and pathways [5]–[9]. The end of the larval phase is marked by the beginning of metamorphosis which consists of a complex reorganization of larval structures. After pupal ecdysis, the pupa undergoes a series of changes in the pigmentation of their compound eyes and of the body until emergence of the imago [10]–[12].

In order to combat microbial infections honey bees, like all insects, rely solely on innate immune reactions that are based on a constitutively active cellular and an inducible humoral immune response [13], [14]. A total of four signalling cascades contribute to innate immunity two of which referred to as Toll and Imd pathways play key roles in the regulation of transcription of target genes coding for antimicrobial peptides (AMPs). They are usually activated by determinants which are conserved in the cell wall of microbes such as lipopolysaccharides or peptidoglycans [14], [15]. The fat body inside the dorsal body cavity of insects is the major immune-responsive tissue for the synthesis and secretion of AMPs in all humoral reactions.

The recent sequencing of the honey bee genome [16] facilitated the comparison with genomic data from other insects and implicated that honey bees possess homologues members of the complex humoral immune response [17]. Moreover, in a comprehensive study Casteels and coworkers discovered and identified a number of bee-specific AMPs in the haemolymph of adult worker bees challenged with *Escherichia coli*, such as apidaecin [18], abaecin [19], hymenoptaecin [20] and defensin 1 [21]. Later, was revealed by RNA interference studies that the expression of hymenoptaecin and abaecin but not of defensin 1 is mediated by the Imd signalling cascade [22]. We have recently studied the defence reactions of honey bee workers and drones and have confirmed the *in vivo* expression of AMPs in the haemolymph after bacterial challenge by qualitative proteomic analyses in combination with mass spectrometry [23], [24].

Although a considerable amount of information is available about humoral immune reactions, little is known about the cellular immune system in honey bees. The cellular immune response comprises wound healing, phagocytosis, nodulation and encapsulation of the intruder. All of these reactions are mediated by the insect blood cells, the haemocytes. They phagocytose bacteria, trap microbes in nodules and encapsulate large parasites [25]. Haemocytes are classified by their morphology and physiology and vary considerably in different insect orders. In Lepidoptera, up to five populations can be identified whereas in Diptera three to four haemocyte types are present [13]. First studies to characterize the haemocytes of honey bees have revealed three populations [26], [27] but their functions in cellular immune responses are unknown. Only nodulation reactions have been studied to some extent in honey bees. It was demonstrated that nodule formation was evoked in newly emerged worker bees artificially infected with freeze-dried *Serratia marcescens* bacteria [28] and in young adult honey bee workers and drones challenged with viable *E. coli* bacteria [24], [29].

Studies of the immunocompetence of honey bees across various life stages and castes have revealed no differences or have disclosed alterations in the defence reactions depending on the assays used for measuring the immune strength [30]–[33]. We have found that drone and worker larvae respond with a strong humoral reaction upon artificial bacterial challenge manifested by the production of bee-specific AMPs. Noteworthy, newly emerged adults – but not larvae – additionally reacted with the up-regulation of several large proteins, among them members of the carboxylesterase family and peptidoglycan recognition proteins [23], [24]. Furthermore, we discovered that the impact of an infection with Acute bee paralysis virus (ABPV) on worker larvae and adult bees differs considerably. Adult bees survive a higher dose of ABPV particles for a longer time post-infection as compared to larvae who additionally respond to viral infection with a quick growth retardation and an extreme change in the appearance of the larval body [29], [34].

In this report, we have expanded our studies on the immune competence of all castes of the honey bee with emphasis on the temporal pattern of cellular and humoral immune responses upon bacterial challenge. Moreover, we have investigated putative defence strategies of honey bee pupae and of long-lived winter bees whose immune competence was altogether unknown to this date. On the one hand, we anticipated that pupae – whose development to the adult bee takes place in sealed brood cells under highly controlled brood temperatures [35] – can cope with a restricted immune system. To our surprise, our studies clearly revealed that worker and drone pupae are totally incapable of activating humoral or cellular immune reactions upon artificial bacterial infections. On the other hand, we found that clustering winter bees maintain important components of the innate immune system and hence are able to combat microbial challenge effectively.

## Results

### Honey Bee Larvae are Able to Cope with Bacterial Infections

We have recently found that *in vitro* reared worker and drone larvae challenged with Escherichia *coli* responded with a prominent humoral reaction 24 and 48 h post-injection as documented by the induced synthesis of the three antimicrobial peptides (AMPs) hymenoptaecin, defensin 1 and abaecin. The presence of AMPs in haemolymph samples was revealed by qualitative proteomic analyses in combination with mass spectrometry and their antimicrobial activity was confirmed in inhibition-zone assays [23], [24].

**Figure 1:**
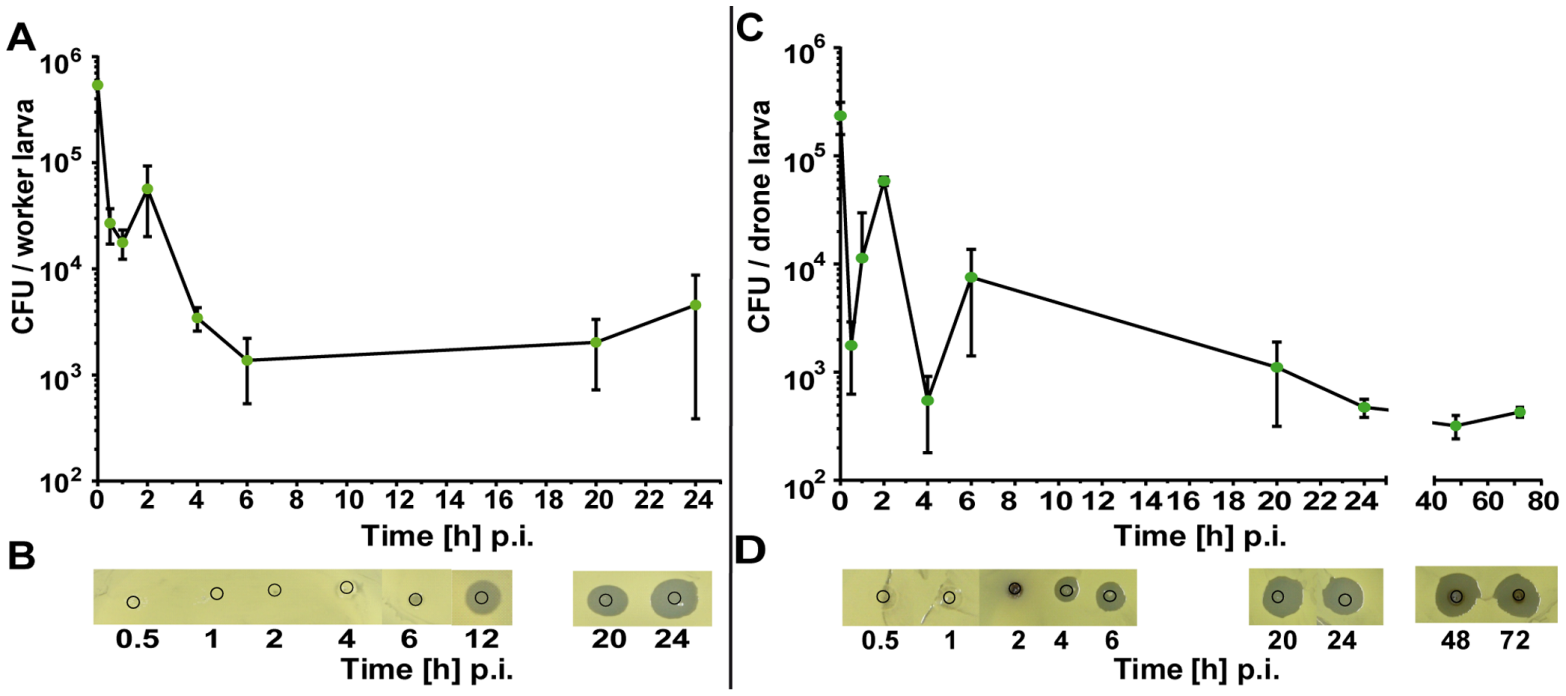
Fate of injected viable *Escherichia coli* bacteria in honey bee larvae. (**A**, **C**) Number of colony-forming units (CFU) recovered from the haemolymph of infected larvae. Four-day-old worker larvae (n = 4) (**A**) and six-day-old drone larvae (n = 3) (**C**) were challenged with ∼10^5^ *E. coli* cells and haemolymph was collected at the indicated times post-injection (p.i.). Aliquots of the haemolymph were spread onto agar plates to estimate the number of surviving bacteria. Each point represents the mean number of CFUs ± standard deviation. Separate analyses of covariance testing for CFUs recovered at different times post-injection revealed significant effects for worker larvae at 0.5 h, 6 h and 24 h p.i. (*p*<0.0001) as well as for drone larvae at 0.5 h (*p* = 0.0108), 24 h, 48 h and 72 h (*p* = 0.0106). (**B**, **D**) Inhibition-zone assay for the detection of antimicrobial activities in the haemolymph of infected larvae. Haemolymph aliquots derived from larvae examined in (**A**, **C**) were applied onto agar plates together with *Micrococcus flavus* as indicator bacteria. The original area of application is encircled.

In the present study, we have clarified the fate of injected viable *E. coli* bacteria in the haemocoel of challenged individuals predominantly at early times, i.e., 0.5 to 6 h post-injection. The initial concentration of *E. coli* bacteria was 5.4±0.7×10^5 ^CFUs per µl for worker larvae (Figure 1A) and 2.4±0.8×10^5 ^CFUs per µl for drone larvae (Figure 1C). The mean concentration of viable *E. coli* bacteria circulating in the larval haemocoel decreased rapidly during the first 4 to 6 h p.i. by more than two orders of magnitude. In worker larvae, the detectable amount of CFUs remained more or less constant between 6 and 24 h p.i. at a level of 2±1.3×10^3^. The number of *E. coli* bacteria detected in drone larvae was reduced to a concentration of 3.7±1.4×10^2^ at 48 h p.i. (Figure 1C). It should be noted that the prepupal stage in drones is reached at the ninth day after hatching [24], whereas the development of worker larvae until the prepupal stage takes only six days [23], explaining the different culture times in Figure 1A and 1C.

The total antimicrobial activity expressed in the haemolymph of larvae challenged with *E. coli* was determined in parallel with the same haemolymph samples as used in Figure 1A and 1C. The inhibition-zone assays revealed that antimicrobial activity is weakly detected in worker larvae 6 h p.i. (Figure 1B), whereas the first occurrence of antimicrobial activity in infected drone larvae was observed as early as 4 h p.i. (Figure 1D). The total amount of antimicrobial activity increased up to 24 h p.i. (Figure 1B) and remained at a constant high level (Figure 1D) as deduced from the size of inhibition zones (Figure 1B, 1D).

### Young Honey Bee Workers are Allround Performers

Newly emerged honey bee workers have been shown to react with a strong humoral immune response to bacterial challenge inducing the same three AMPs hymenoptaecin, defensin 1 and abaecin as detected in worker larvae [23]. In contrast to larvae, we found that in adult workers several immune-responsive proteins were additionally induced or up-regulated after *E. coli* infection [23], [36]. Moreover, it was demonstrated that in young adult worker bees challenged with bacteria nodulation reactions are evoked [28], [29], a specific cellular defence mechanism of insects [37].

**Figure 2:**
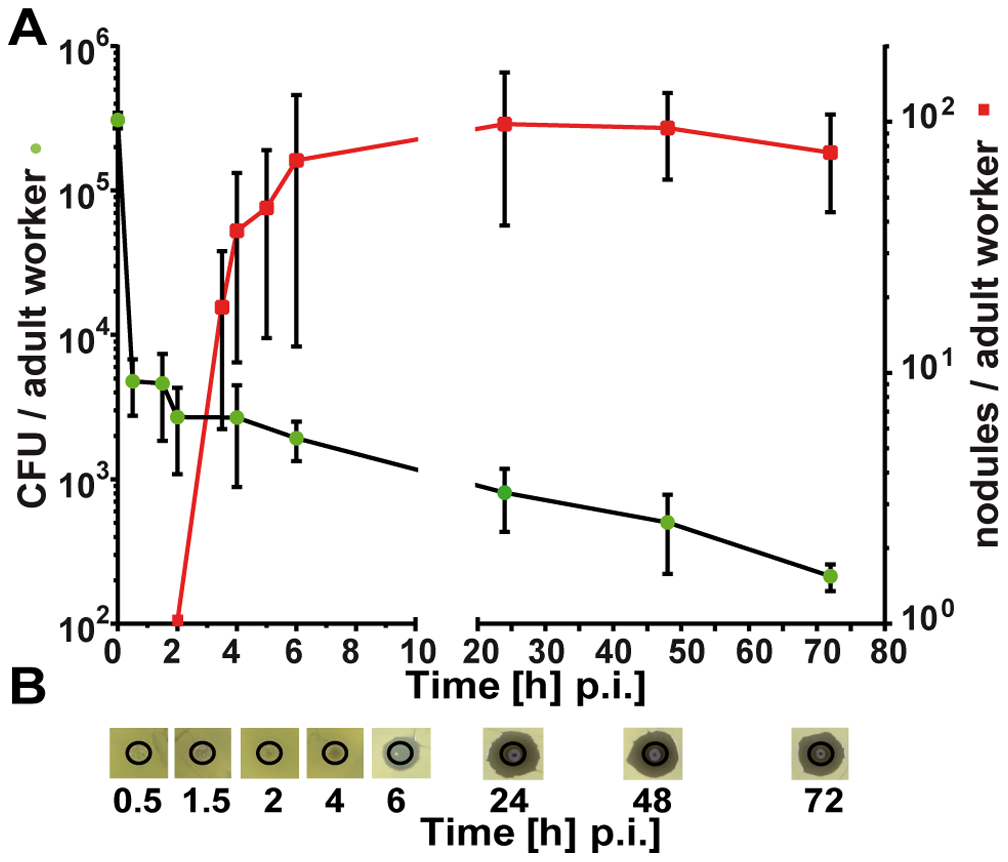
Fate of injected viable *E. coli* bacteria in honey bee adult workers. (**A**) Number of colony-forming units (CFU) recovered from the haemolymph (left y-axis, •) and nodule formation in the haemocoel of infected workers (right y-axis, ▪). Newly emerged worker bees (1d) were challenged with ∼10^5^ *E. coli* cells and were subsequently divided into two groups: haemolymph was recovered from one batch of individual workers (n = 4–5) at the indicated times (p.i.) and aliquots were spread on nutrient agar plates in order to determine the number of CFUs and the number of melanised nodules per individual worker bee (n = 5–12) was assessed of the second group. The error bars represent the standard deviation. (**B**) Inhibition-zone assay for the detection of antimicrobial activities in the haemolymph of adult worker bees after infection with *E. coli* at the indicated times.

In a comprehensive study, we have now compared the time-dependent formation of nodules, the fate of injected bacteria (colony-forming units per bee) and the induction of antimicrobial activity upon artificial infection with *E. coli* in parallel assays. We performed two independent series of experiments whose results were essentially in accordance so that we have combined the data (Figure 2). The initial concentration of *E. coli* bacteria was 3±0.4×10^5^ CFUs per µl which was injected into each of about 70 newly emerged worker bees per series. Half of them were employed for assessing nodulation reactions and the other half was used to measure the number of colony forming units (CFUs) and antimicrobial activity in the haemolymph extracted from challenged bees. The adult workers were kept in groups of 10 individuals in small cages before analysis at the indicated times post-injection.

The number of detectable *E. coli* bacteria circulating in the haemocoel of adult worker bees was reduced rapidly within the first 30 min p.i. from 3±0.4×10^5^ to 4.8±2×10^3^ and declined further to 2.1±0.5 x10^2^ at 72 h p.i. (Figure 2A). The first nodules could be identified between 3 and 4 h p.i. (18.3±12.3 nodules/bee). Subsequently, we recorded an increased number of nodules up to 6 h p.i. (70.2±57.4 nodules/bee), an almost constant level between 24 and 48 h p.i. (94.4±35.6 nodules/bee at 48 h p.i.) and a slight decline at 72 h p.i. (75.2±31.6 nodules/bee). Antimicrobial activity in the haemolymph of young adult workers challenged with *E. coli* was first detectable at 6 h p.i., increased up to 24 h p.i. and remained constant until 72 h p.i. (Figure 2B).

### Winter Bees are Well-prepared for Bacterial Challenge

Honey bees survive the winter as a complete functional colony with about only 1/5 to 1/10 of the total population in summer. The winter bees cling together in a dense cluster and keep themselves warm by vibrating their wing muscles [1]. We asked ourselves whether winter bees preserve the potential to activate various components of the innate immune system inspite of the fact that a) external infections of the bee cluster are rather unlikely during the winter season and that b) any immune reaction is coupled to high energy costs [38]. In preliminary experiments, we had observed that humoral immune reactions are induced in winter bees as strongly as in summer bees [39]. We have now examined in more detail the ability of winter bees to combat bacterial infections by humoral and/or cellular immune reactions.

**Figure 3:**
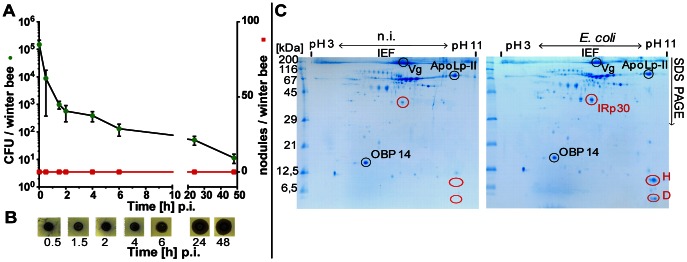
Fate of injected viable *E. coli* bacteria in winter bees. Worker bees were removed from the hive during the winter season (January/February, 2010) and kept in small cages in an incubator at 26°C. They were artificially infected with ∼10^5^ viable *E. coli* cells and divided into two groups. (**A**) Number of CFUs recovered from the haemolymph of winter bees (n = 4) at the indicated times p.i. (left y-axis, •) and number of melanised nodules per individual winter bee (n = 15) (right y-axis ▪). (**B**) Inhibition-zone assay for the detection of antimicrobial activities in the haemolymph of winter bees after infection with *E. coli* at the indicated times. Aliquots of the samples examined in (A) were applied onto agar plates together with *M. flavus* as indicator bacteria. Inhibition-zones looked brownish in the area of sample application possibly because haemolymph from older worker bees contains small amounts of melanin [29]. (**C**) Two –dimensional gel electrophoretic analyses of soluble proteins present in the haemolymph of winter bees at 24 h post-infection with 10^5^ *E. coli* bacteria. For comparison, the protein pattern of non-infected (n.i.) individuals is shown to the left. Proteins were separated by isoelectric focusing (IEF) in the first dimension and by SDS-PAGE in the second dimension. Encircled spots were excised and the proteins were identified by subsequent MS/MS analyses as described [24]. Differentially expressed immune-responsive proteins are marked by red circles. Vg, vitellogenin (gi|58585104); ApoLpII, apolipophorin (gi|66513966); OBP14, odorant binding protein 14 (gi|94158822); IRp30 (gi|66507096); H, hymenoptaecin (gi|58585174), D, defensin 1 (gi|37703274). Gels were stained with Coomassie Brilliant Blue G250.

Unexpectedly, winter bees were totally unable to respond with the formation of nodules upon *E. coli* infection (Figure 3A). We have carried out these studies in three independent series of experiments during two winter seasons, but never detected any nodules at any time post-injection.

However, we discovered a rapid reduction of viable *E. coli* bacteria in the haemolymph of challenged winter bees which was even faster and more effective then observed in summer bees (Figures 2, 3). The initial concentration of *E. coli* was 1.5±0.6×10^5^ CFUs/µl and the number of CFUs was as low as 1.3±0.6×10^2^ at 6 h post-injection. At further incubation times up to 48 h p.i., viable *E. coli* bacteria were hardly detectable any more, i.e., 11±4 CFUs/winter bee (Figure 3A). Antimicrobial activity in the haemolymph was first visible at 6 h p.i. and reached a maximum level between 24 and 48 h p.i. (Figure 3B). The induction of true antimicrobial peptides was confirmed by two-dimensional gel electrophoretic analyses of the haemolymph proteins which revealed the expression of hymenoptaecin and defensin 1 upon *E. coli* infection 24 h p.i. (Figure 3C). A major immune-responsive protein, named IRp30, was found to be expressed in newly emerged adult workers [23], [36]. Its synthesis is also up-regulated in winter bees as previously disclosed [36] and shown in Figure 3C.

### Drones are Protected by a Powerful Innate Immune System

In temperate regions, honey bee colonies harbour approximately 2000 drones during a short period of the summer season (in addition to about 50,000 workers). We have recently shown that newly emerged adult drones express the same array of immune reactions as honey bee workers, i.e., a strong humoral immune response and a pronounced accumulation of nodules upon *E. coli* infection [24].

**Figure 4:**
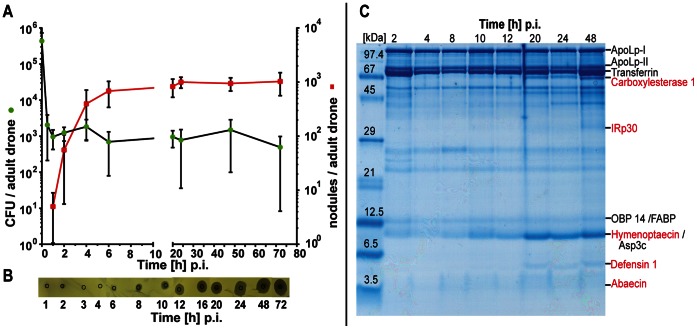
Fate of injected viable *E. coli* bacteria in honey bee adult drones. (**A**) Number of CFUs recovered from the haemolymph (left y-axis •) and nodule formation in the haemocoel of infected drones (right y-axis ▪). Newly emerged drones (1 d) were challenged with ∼10^5^ *E. coli* bacteria and were subsequently divided into two groups: haemolymph was recovered from one batch of individual drones (n = 6) at the indicated times (p.i.) and aliquots were spread on nutrient agar plates in order to determine the number of CFUs and the number of melanised nodules per individual drone (n = 5–11) was assessed of the second group. The error bars represent the standard deviation. (**B**) Inhibition-zone assay for the detection of antimicrobial activities in the haemolymph of adult drones after infection with *E. coli* at the indicated times. (**C**) Gel electrophoretic analyses of proteins present in the haemolymph of adult drones at the indicated times post-injection of ∼10^5^ *E. coli* bacteria. Aliquots of haemolymph samples were applied onto a 15% polyacrylamide/0.1% SDS gel [83]. Gels were stained with Coomassie Brilliant Blue G250. The induced antimicrobial peptides and immune-responsive proteins are indicated in red.

Similarly, as described in the second chapter, we performed a comprehensive study with adult drones encompassing time course experiments of humoral and cellular immune reactions after bacterial challenge. About 10^5^ *E. coli* bacteria were injected into each of 70 newly emerged adult drones per series. Half of them were employed for assessing nodulation reactions and the other half was used to collect haemolymph at indicated times post-injection. The haemolymph samples served as sources to determine a) the number of surviving bacteria, b) the total antimicrobial activity by means of zone-inhibition assays and c) the specifically induced AMPs by gel electrophoretic analyses. The number of detectable *E. coli* bacteria in the haemolymph declined rapidly from 4.4±0.3×10^5^ to 9.6±5.3×10^2^ CFUs/adult drone within the first 60 min p.i. and thereafter remained essentially constant until 72 h post-injection (Figure 4A). The first nodules in infected adult drones were identified at 1 h p.i. (5±4 nodules per drone). The detectable number of melanised nodules then increased rapidly, varying only slightly between 20 h (827±305) and 72 h p.i. (1025±463 nodules/adult drone) as shown in Figure 4A.

Antimicrobial activity in the haemolymph of young adult drones challenged with *E. coli* was first recorded at 8 h post-injection. Thereafter the total amount of antimicrobial activity increased steadily up to 20 h p.i. and then remained unaltered (Figure 4B). The induction of antimicrobial compounds as demonstrated by zone-inhibition assays was confirmed by proteomic analyses of the same haemolymph samples which revealed the time-dependent expression of the known AMPs hymenoptaecin, defensin 1 and abaecin (Figure 4C).

### The Immune Strength of Honey Bee Queens Matches that of Worker Bees

Queens of honey bees differ from sterile female workers in many aspects. For instance, they are twice as large, develop faster from egg to adult, are adapted anatomically to the high output of egg production and can live for several years [11], [40]. The caste differentiation process depends on quantity and quality of larval food supplied to queens by nurse bees [4]. A mated queen remains in the hive where she is provided with food and receives special care by attendant workers during her whole life-span, in the course of which she is exposed to pathogens transmitted by the workers surrounding her.

**Figure 5:**
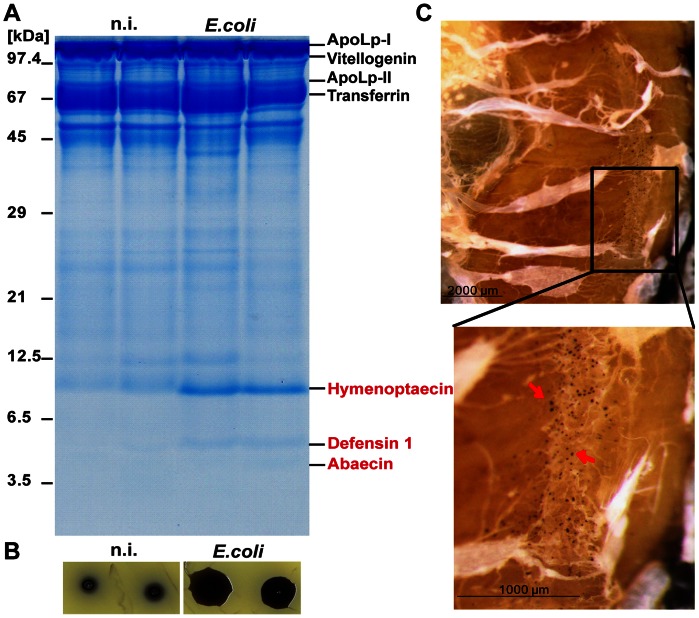
Immune reactions of honey bee queens. (**A**) Characterization of induced antimicrobial peptides (AMPs) in honey bee queens by gel electrophoretic analyses of haemolymph proteins. Two young queens (2 days old) were left untreated and two queens were challenged with ∼10^5^ viable *E. coli* bacteria. Single queens were kept in small cages with five worker bees in an incubator at 35°C and 70% relative humidity. They were supplied with Apifonda *ad libitum*. The haemolymph was collected 24 h post-injection of each individual. Aliquots of these samples were applied onto a 15% polyacrylamide/0.1% SDS gel [83]. Gels were stained with Coomassie Brilliant Blue G250. Induced AMPs are indicated in red. (**B**) Inhibition-zone assay for the detection of antimicrobial activities in the haemolymph of queens challenged with *E. coli*. Aliquots of the same haemolymph samples as in (A) were applied onto agar plates together with *Micrococcus flavus* as indicator bacteria. (**C**) Nodulation reaction of queens. A total of 8 young queens (7 days old) were challenged with 10^5^ viable *E. coli* bacteria. Upon 24 h p.i., abdominal tergits were removed and the body cavity of queens was exposed followed by inspection for the presence of melanised nodules (indicated by red arrows). Photomicrographs were taken with an Olympus SZX7 stereomicroscope equipped with an Olympus UC30 camera. Magnification was x20 for the complete abdomen and x32 for the detailed view.

We have examined the defence systems of young adult queens (2 to 7 days old) and have found that they react to challenge with *E. coli* bacteria with a strong humoral response as seen by the synthesis of specific AMPs in the haemolymph (Figure 5A) and by the induction of antimicrobial compounds deduced from inhibition-zone assays (Figure 5B). Moreover, we have also recorded the formation of nodules in the haemocoel of young queens (n = 8) amounting to 179±107 nodules/queen (Figure 5C). Furthermore, we have previously demonstrated that some immune-responsive proteins such as carboxylesterase (CE 1) and IRp30 are as readily induced in queens as in workers upon bacterial challenge [36].

### Bee Pupae are Incapable of Activating Defence Reactions

Workers and drones proceed through five larval stages albeit of different lengths. Worker larvae need five, whereas drone larvae require nine days until the prepupal stage [23], [24], [40]. At this point, the larvae stretch out and begin to spin themselves a cocoon within the cell. At the same time the cell is sealed with a wax lid by worker bees [1]. After a period of about 12 to 13 (workers) or 14 to 15 days (drones), adult individuals emerge from the cells by opening the cell lid from within. The transformation of pupae to the imago becomes visible by the appearance of three major body parts that are equivalent to head, thorax and abdomen of adults and the further development is accompanied by a gradual darkening of the whole body. A characteristic feature of the successive development of pupae is the pigmentation of their eyes changing from an opalescent white to a pink, dark red and finally brownish colour [10]–[12].

**Figure 6:**
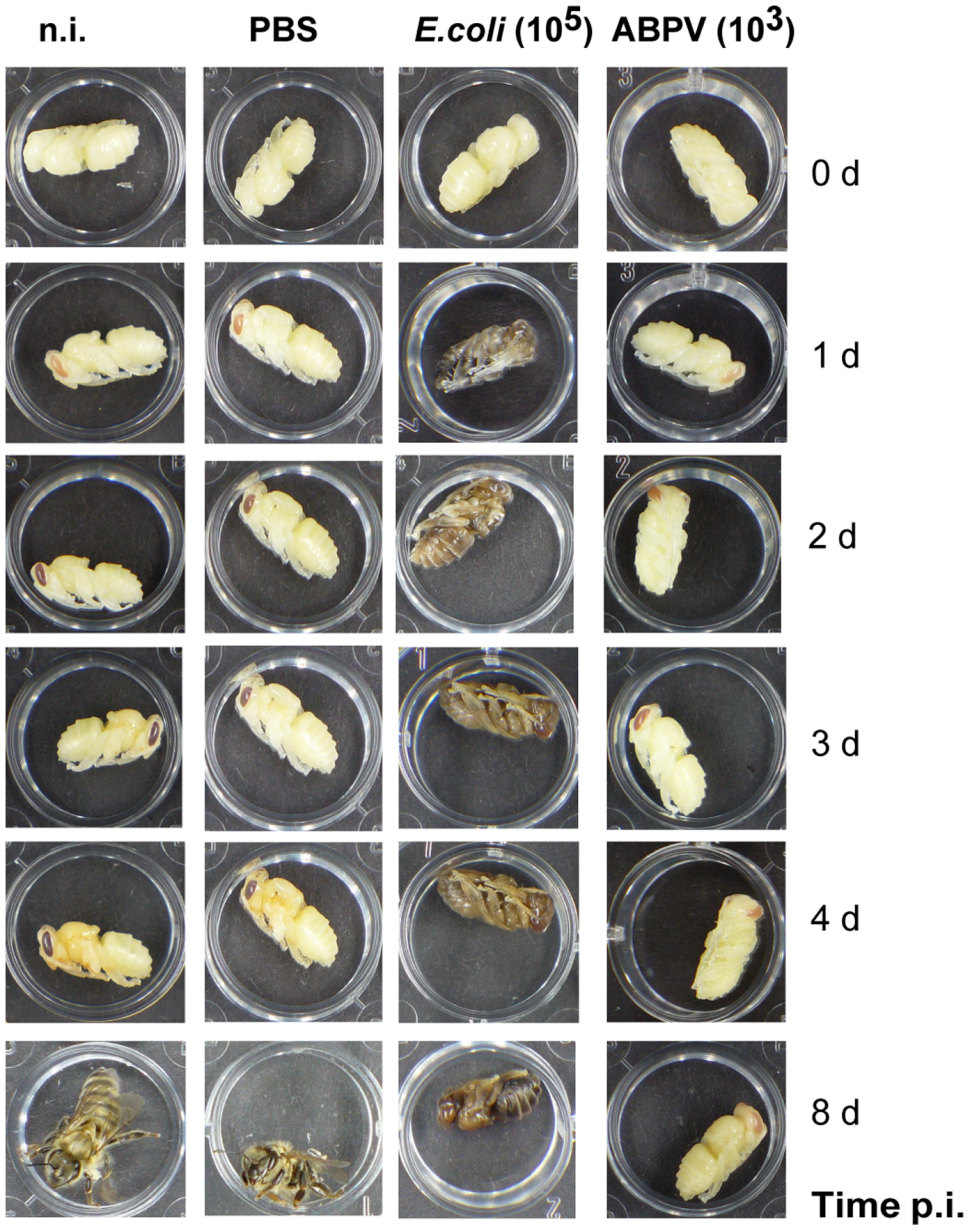
Comparison of *in vitro* cultured worker pupae after aseptic and septic wounding. White-eyed worker pupae were left untreated (n.i.), challenged with PBS, 10^5^ *E. coli* bacteria or with 10^3^ ABPV particles. The pupae were kept in tissue culture plates in an incubator at 35°C. Their development was recorded until the emergence of worker bees in the control group (n.i.).

Honey bee pupae are routinely employed for virus propagation, e.g., the Acute bee paralysis virus (ABPV). For this purpose, an ABPV suspension is injected into the haemocoel of white-eyed worker pupae, the infected pupae are maintained in an incubator at 35°C for three days and subsequently used for the preparation of purified virus [29]. We have examined the impact of ABPV infection on the appearance of pupae and found that during the first two days after ABPV infection pupae developed similarly as the controls, exhibiting a change from white to red eyes. However, the development of ABPV-infected pupae ceased entirely at the red-eyed pupal stage in contrast to the control individuals whose eye colour changed to dark brown accompanied by a gradual pigmentation of the thorax (Figure 6).

For comparison, we infected white-eyed worker pupae with about 10^5^ *E. coli* bacteria per pupa, a concentration that had been shown to have no deleterious impact on larvae or adult bees [29], [34]. To our great surprise, the pupae did not survive *E. coli* infections at all, comprising a gray-brownish appearance and a soft constitution already 24 h post-injection. Upon further cultivation, the *E. coli*-infected pupae collapsed totally (Figure 6). In order to rule out that pupae succumbed to challenge with *E. coli* due to damage by the employed procedure we injected volumes of 1 µl of phosphate –buffered saline (PBS) into the pupae and observed no deleterious effects during their subsequent development (Figure 6).

**Figure 7:**
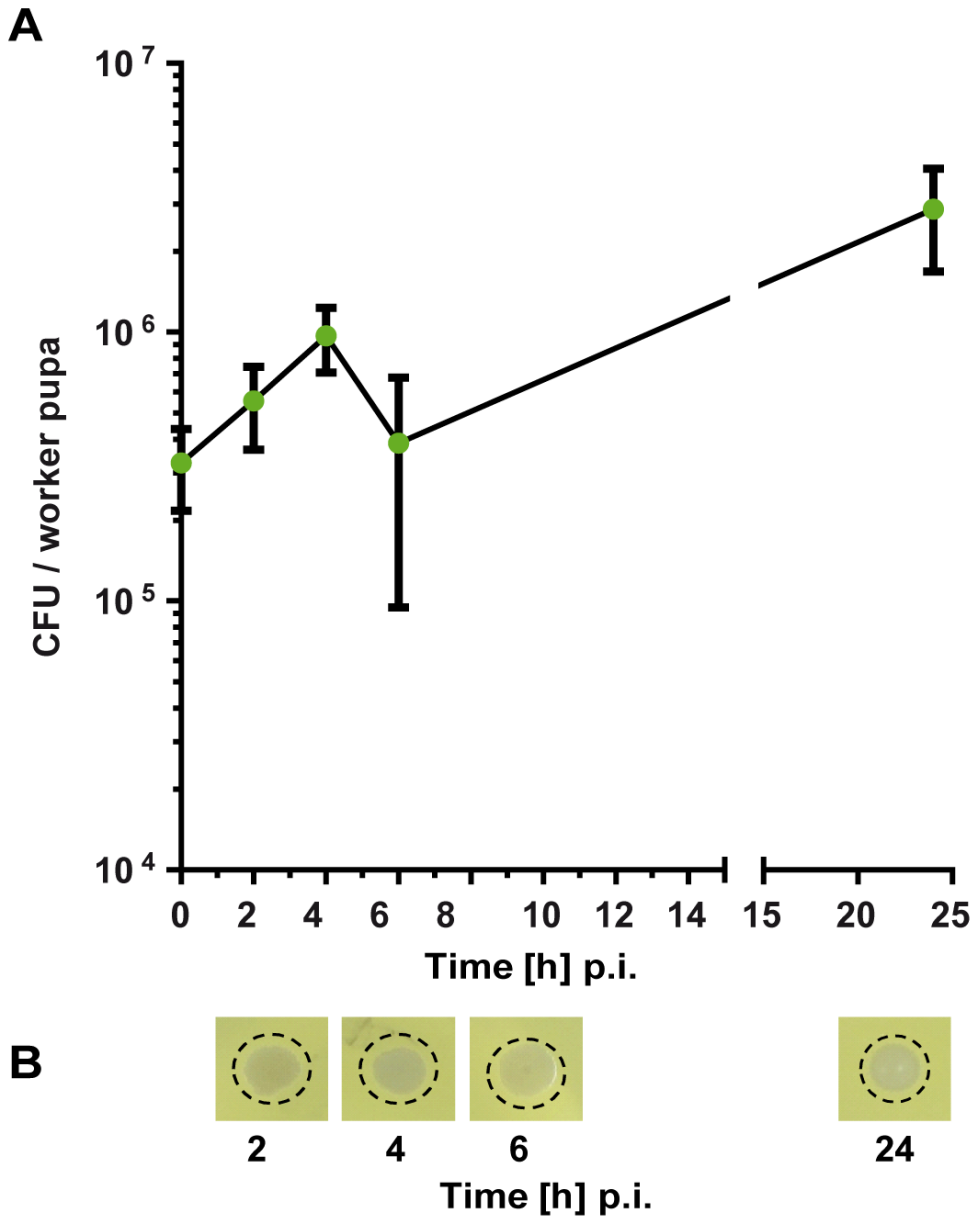
Fate of injected viable *E. coli* bacteria in honey bee worker pupae. White-eyed pupae were collected from sealed combs, transferred into empty tissue culture plates and subsequently kept in an incubator at 35°C and 70% relative humidity. They were challenged with *E. coli* bacteria as demonstrated in Figure 6. (**A**) Number of CFUs recovered from the haemolymph of worker pupae (n = 3) post-infection with ∼10^5^ *E. coli* cells at the indicated times. The error bars represent the standard deviation. (**B**) Inhibition-zone assay for the detection of antimicrobial activities in the haemolymph of worker pupae infected with *E. coli*. Aliquots of the samples were applied onto agar plates together with *M. flavus* as indicator bacteria. The area which is covered with newly grown bacteria colonies is indicated by a dotted ring.

These observations prompted us to study humoral and cellular immune responses of pupae in more detail. We injected 3.3±1.1×10^5^ *E. coli* bacteria into the haemocoel of each of about 50 white-eyed pupae and collected haemolymph samples at intervals between 2 and 24 h later. We discovered a gradual increase of viable *E. coli* bacteria up to 2.9±1.2×10^6 ^CFUs/worker pupa at 24 h p.i. (Figure 7A) quite in contrast to the fate of bacteria in infected larvae and adult bees where we had observed a rapid decline of CFUs within the first 6 h p.i. (Figures 1–4). Additionally, antimicrobial activity was not detectable in the haemolymph of worker pupae challenged with *E. coli* up to 24 h post-injection. We observed tiny white colonies in the area where we had applied an aliquot of the corresponding haemolymph sample onto the test plates instead of a clear zone (Figure 7B).

**Figure 8:**
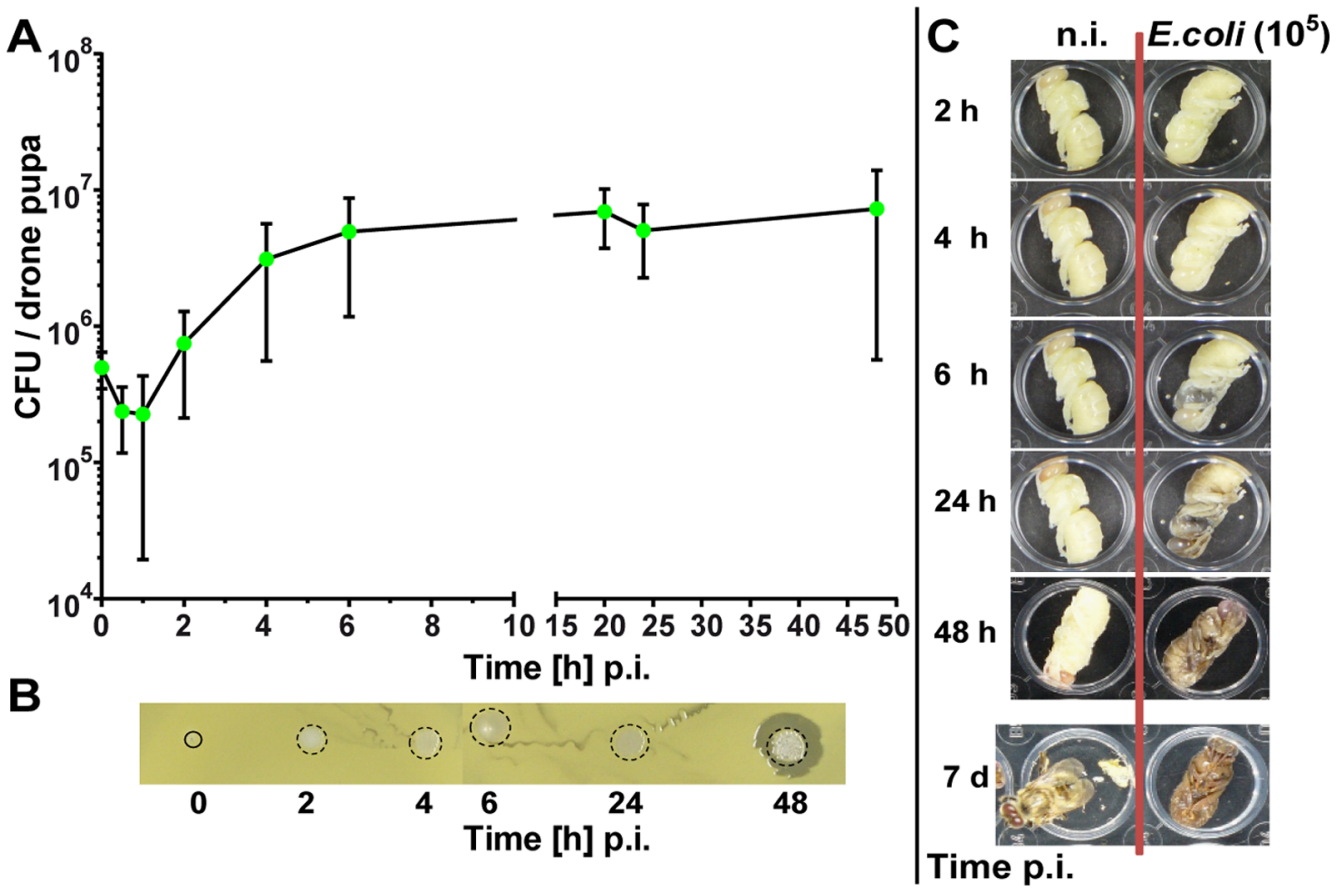
Fate of injected viable *E. coli* bacteria in honey bee drone pupae challenged with a high dose. White-eyed drone pupae were collected from sealed combs, transferred into empty tissue culture plates and subsequently kept in an incubator at 35°C and 70% relative humidity. They were left either untreated (n.i.) or challenged with *E. coli*. (**A**) Number of CFUs recovered from the haemolymph of drone pupae (n = 3–4) at the indicated times post-infection with ∼10^5^ *E. coli* bacteria. The error bars represent the standard deviation. (**B**) Inhibition-zone assay for the detection of antimicrobial activities in the haemolymph of drone pupae infected with *E. coli*. Aliquots of the samples were applied onto agar plates together with *M. flavus* as indicator bacteria. The area which is covered with newly grown bacteria colonies is indicated by a dotted ring. (**C**) Development of *in* *vitro* cultured white-eyed drone pupae within 7 days post-injection of ∼10^5^ E. coli bacteria. For comparison, the development of non-infected control pupae to the imago is shown to the left.

In a further series of experiments, we also studied the fate of bacteria in drone pupae. We injected 4.9±1.4×10^5^ *E. coli* bacteria into each of about 40 drone pupae per series and collected haemolymph samples between 0.5 and 48 h post-injection. Within the first hour p.i., we observed a weak decline of viable *E. coli* bacteria followed by a rapid proliferation of bacteria to a concentration of 7.3±6.8×10^6^ CFUs/drone pupa upon 48 h p.i. (Figure 8A). In the inhibition-zone assay, we did not detect any antimicrobial activity up to 24 h post-injection of *E. coli* bacteria, but rather observed tiny colonies instead of clear zones at the areas of haemolymph application. However, a clear zone was observed surrounding the bacterial colonies in haemolymph samples collected 48 h p.i. from infected drone pupae (Figure 8B), suggesting that a humoral response was after all induced albeit too late to have any impact on fighting bacterial infections. White-eyed control drone pupae developed normally, i.e., 48 h after the onset of *in vitro* cultivation red eyes were manifested and 7 days later the first adult drones emerged. In contrast, drone pupae infected with 10^5^ *E. coli* bacteria/pupa at the white-eyed stage adopted a grayish complexion as soon as 6 h p.i. and collapsed between 24 and 48 h p.i., showing a dark brownish appearance (Figure 8C).

**Figure 9:**
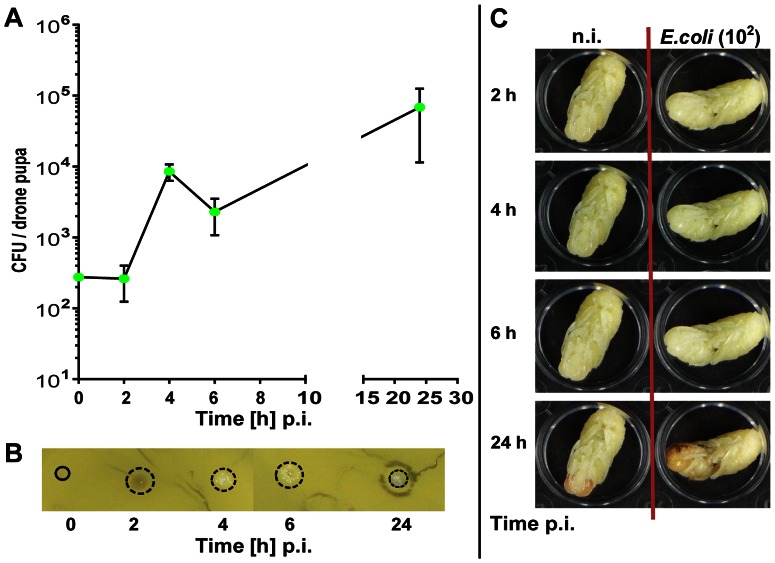
Fate of injected viable *E. coli* bacteria in honey bee drone pupae challenged with a low dose. White-eyed drone pupae were collected from sealed combs, transferred into empty tissue culture plates and subsequently kept in an incubator at 35°C and 70% relative humidity. They were left either untreated (n.i.) or challenged with *E. coli*. (**A**) Number of CFUs recovered from the haemolymph of drone pupae (n = 3–4) at the indicated times post-infection with ∼10^2^ *E. coli* bacteria. The error bars represent the standard deviation. (**B**) Inhibition-zone assay for the detection of antimicrobial activities in the haemolymph of drone pupae infected with *E. coli*. Aliquots of the samples were applied onto agar plates together with *M. flavus* as indicator bacteria. The area which is covered with newly grown bacteria colonies is indicated by a dotted ring. (**C**) Development of *in* *vitro* cultured white-eyed drone pupae within 24 h post-injection of ∼10^2^ E. coli bacteria. For comparison, the development of non-infected control pupae is shown to the left.

Injection of only 2.8±0.4×10^2^ *E. coli* bacteria into white-eyed drone pupae also resulted in the death of all infected pupae, albeit with some delay as compared to pupae challenged with about 10^5^ *E. coli* bacteria. The first visible signs of premature grayish colouring of the pupae cuticle appeared at 24 h p.i. (Figure 9C) and was accompanied by an increase of the *E. coli* concentration in the haemocoel to 6.8±5×10^4^ CFU/pupa and the absence of clear inhibition zones (Figure 9A, 9B). Similarly, injection of low doses of *E. coli* (about 10^3^ CFUs/pupa) into white-eyed worker pupae culminated in an increase of viable *E. coli* bacteria by two orders of magnitude in the pupal body cavity (not shown).

## Discussion

Within the last two decades, honey bees have attracted special attention because of the catastrophic colony losses worldwide. In Europe, the problems started with the introduction of the ectoparasitic mite *Varroa destructor* leading to a newly acquired virulence of several bee viruses [41].In the USA, the colony collapse disorder (CCD), a phenomenon characterized by a yet unexplained hive abandonment of the total worker honey bee population has resulted in a loss of 50 to 90% of colonies [42], [43]. In the context of increased mortality of individual honey bees and/or losses of whole colonies, it has often been suggested that their immune competence is impaired or even suppressed [44], [45]. This raises the question about the definition of global immune competence and the parameters by which it is ascertained. In fact, quite different assessments are taken to determine the immune strength of individual bees, from haemocyte counting, fat body quantification, encapsulation response, phenoloxidase activities, inhibition-zone assays to studies of the transcriptional up-regulation of genes involved in bee-specific humoral immune reactions [17], [30]–[33], [38], [46], [47]. As far as honey bees are concerned, the definition of immune competence is further impeded by the existence of different castes and different developmental stages varying throughout summer and winter seasons. In a comprehensive study, we have examined and compared the immunocompetence of drone and worker bees at all important life stages employing a broad array of assays that represent various aspects of cellular and humoral reactions. Our results clearly demonstrate that honey bees have adapted to microbial infections quite reasonably according to their needs.

To make sure that we studied the response of healthy individuals upon artificial infections we took several precautions: (a) healthy bee colonies were selected that were free of chalkbrood and American foulbrood. Infestation with the *Varroa* mite had been kept at a low level by a professional beekeeper; (b) *in vitro* cultivation and artificial infection of worker and drone larvae facilitated studies under sterile conditions [23], [24]; (c) worker and drone pupae were kept in sterile tissue culture plates in which development proceeded normally (Figures 6, 8, 9) and (d) newly emerged adults were kept in clean, small boxes. In fact, we never detected any antimicrobial activity in control haemolymph samples from naive individuals as verified by inhibition-zone assays [23], [24], [29].

Winners in our studies are newly emerged adult worker bees and drones. They were able to activate all known cellular and humoral defence reactions (Figures 2, 4). It appears reasonable that not only female workers but also male drones are equipped with the same repertory of defence reactions, since male reproductive success is a “major driver of natural selection in honey bees” [48]contributing to the overall colony fitness and since a colony has invested a lot of resources into the rearing and maintenance of a certain number of drones during the reproductive period [35], [49]. The number of viable bacteria circulating in the haemolymph of challenged bees declined rapidly by more than two orders of magnitude within 30 to 60 min post-injection of about 10^5^ *E. coli* bacteria coinciding with a rapid increase of detectable melanised nodules. Antimicrobial activity was first ascertained around 6 h post-injection, increased up to 24 h p.i. and then remained constant during the observation period. These two temporal patterns of immune reactions very likely represent the rapid constitutively active cellular immune response comprising mainly phagocytosis and nodulation and the induced humoral immune reaction that ultimately leads to the production of various antimicrobial peptides. Such temporal dynamics towards microbial invasions has been reported elsewhere [50]–[53]. Korner and Schmid-Hempel [51] found that workers of the bumble bee *Bombus terrestris* reacted to challenge with immune elicitors with a complex pattern of enhanced phenoloxidase activity that was correlated with haemocyte counts. Antibacterial activity, on the other hand, was not detected immediately after infection, but increased between 2 and 24 h post-challenge. The susceptibility of *Manduca sexta* larvae to infection by several Gram-negative pathogenic and non-pathogenic bacteria has been investigated by Dunn and Drake [50]. The concentration of all examined bacteria was reduced significantly during the first hour followed by a second phase that either led to the complete elimination of viable bacteria or to bacterial multiplication and host death depending on the strain. These authors discuss that phagocytosis and/or nodulation reactions are responsible for the rapid reduction of all types of bacteria in the first period but that *M. sexta* larvae tolerate non-pathogenic bacteria such as *E. coli* or low doses of *Pseudomonas aeruginosa* in the second phase because these bacteria are sensitive to induced humoral antimicrobial factors whereas pathogenic bacteria are not. Haine and colleagues [53] have explored the ability of the beetle *Tenebrio molitor* to inactivate a large dose of injected *Staphylocuccus aureus* bacteria from the haemocoel and observed a very rapid clearance of *S. aureus* within the first hour. Induced antimicrobial activity started to increase in the haemolymph of challenged beetles after most bacteria had been removed and remained elevated until 28 days after the initial infection. Further experiments revealed that the function of induced microbial compounds late in infection is to protect the host against bacteria that have survived the cellular immune response.

A unique feature of honey bee workers is their diversity in longevity. Summer bees usually live up to 6 weeks whereas the life span of winter bees varies from 6 to 9 months [3], [54]. Winter bees dońt hibernate but stay in a cluster inside the hive mainly engaged in thermoregulation. Insulation of the cluster is achieved by tightly packed mantle bees whilst bees inside the cluster actively produce heat by shivering with their flight muscles [2]. This endothermic heat production is a highly energy-consuming process and relies mostly on honey reserves in a bee colony [1]. Some physiological properties distinguish winter from young summer bees. Winter bees have high titres of the yolk protein vitellogenin which is produced and secreted by the fat body and is thought to protect bees from oxidative cellular damage in addition to other multiple functions [55]. Furthermore, winter bees have an enlarged fat body with a high content of lipids, glycogen and protein and they have a large number of haemocytes [56]. Hence, considering the needs for an efficient cellular and humoral immune response winter bees are basically well equipped to initiate these systems. Without doubt, humoral reactions are induced in winter bees upon artificial infections with *E. coli*. We detected antimicrobial activity in the haemolymph of challenged individuals and identified the two major bee-specific AMPs hymenoptaecin and defensin 1 (Figure 3). Additionally, we observed a fast decrease of viable *E. coli* bacteria by two orders of magnitude within the first two hours post-injection implying a rapid cellular response. The clearance of bacteria from the haemocoel continued until 48 h p.i. at which time almost all viable bacteria appeared to be eliminated (Figure 3A). Despite the effective overall immune response, winter bees are unable to produce melanotic nodules in response to bacterial infections, a dominant cellular defence reaction in most insects [57]. It has been pointed out by Bedick et al. [28] that nodulation reactions might not occur in all phases of insect life cycles, deduced from their findings that honey bee foragers did not evoke any nodules upon septic infections. We cannot fully support this observation because we detected nodules in the haemocoel of forager bees (10.4±9.8; n = 20) as well as of 9 days old hive bees (13±9; n = 10 ) 24 h post-injection of 10^5^ *E. coli* bacteria albeit at a reduced amount as compared to newly emerged worker bees (98±59; n = 14). Hence, there is a clear tendency that honey bee workers with increasing age lose the capacity to produce visible nodules, culminating in the complete absence of nodulation reactions in winter bees. The reasons for this phenomenon are not known. It has been previously demonstrated by Stanley-Samuelson et al. [58] that nodulation is mediated by eicosanoids and that the major elements of an eicosanoid system are present in the fat body and in haemocytes [57], tissues and cells that are abundant in winter bees. Eicosanoids are synthesized from fatty acids, mainly from arachidonic acid that is subsequently metabolized via three different pathways to the final products such as prostaglandins [57]–[59]. We can only speculate that the biosynthetic pathway of eicosanoids is impaired in old workers and in winter bees, possibly because of some deleterious effect of a constitutive eicosanoid synthesis.

Besides cellular and humoral reactions, the activation of prophenoloxidase (proPO) by a serine protease cascade resulting in the transient synthesis of quinones and melanin is a prerequisite for multiple immune processes such as wound healing, phagocytosis and nodulation [14], [60]. Schmid et al. [31] found that in worker bees PO activity increased with age, reaching a plateau within the first week of adult life and remaining constant at a high level until the age of 24 days. Although we have confirmed the presence of the prophenoloxidase zymogen in the haemolymph of winter bees by gel electrophoretic analyses in combination with mass spectrometry (not shown), we have not measured active phenoloxidase so that it remains open whether proPO is converted to PO after bacterial challenge. In summary, we can state that long-lived honey bee workers essentially maintain their full capacity to activate cellular and humoral immune reactions. This fitness of winter bees on the level of immunocompetence is mirrored by a fitness of cognitive functions. Thus, it was shown by Behrends and Scheiner [61] that winter bees did not display an age-related decline in associative learning or discrimination abilities despite their high age. Considering the various activities of honey bee workers during the winter season that involves thermoregulation, queen attendance and even brood rearing in warm phases within the hive and sporadic cleansing flights outside the hive, it is reasonable that winter bees are well prepared to combat any microbial attack they might encounter.

Apart from adult drone and worker bees, we have examined how honey bee larvae react to bacterial infections. Larvae that are constantly supplied in their open brood cells with a diet produced by nurse bees may encounter microbial infections via two principal ways: (1) by ingestions of contaminated diet and (2) by external injury leading to infections of the haemocoel. As shown in this report and earlier studies [23], [24] worker and drone larvae are well prepared to cope with bacterial infections that invade the haemocoel. Both, humoral and cellular immune reactions were initiated in 4–5 days old worker and 6–9 days old drone larvae, respectively, upon challenge with *E. coli* to a level comparable to that of young adult individuals (Figures 1, 2, 4) – with one restriction: The formation of nodules is impaired in honey bee larvae as recently demonstrated [24].

One of the most disastrous bacterial diseases of honey bees is the American foulbrood (AFB) that is caused by the spore-forming bacteria *Paenibacillus larvae*. Only larvae are susceptible to AFB and infection is established by ingestion of spore-contaminated larval food. The spores germinate and a massive proliferation of vegetative *P. larvae* bacteria takes place in the midgut prior to the penetration of the gut epithelium [62]. Consequently, the haemocoel of challenged larvae is flooded with an extremely high dose of *P. larvae* that might demand too much from the humoral and cellular immune system at least from very young larvae. This assumption is supported by the observation that only first instar larvae are highly susceptible to AFB infection whereas honey bee larvae older than two days become resistant to infection [62]–[64]. The age-dependent susceptibility of larvae has been attributed to the age-dependent development and composition of the peritrophic matrix that represents a barrier for the *P. larvae* bacteria to reach the gut epithelium [62]. Furthermore, the fat body being the major tissue for the synthesis of antimicrobial compounds might be too small in first instar larvae to produce sufficient amounts of AMPs. Transcription studies by Evans [65] revealed higher levels of abaecin and defensin 1 in worker larvae exposed to *P. larvae* suggesting that honey bee larvae principally can mount an immune response to this pathogen. In this context it should be mentioned that royalisin contained in royal jelly [66] and identical to the known AMP defensin 1 [67] has been shown to express inhibitory effects against *P. larvae* [68] as well as other Gram-positive bacteria [66].

The loser of all examined life stages of honey bees are the pupae. To our great surprise, worker and drone pupae were completely unable to activate cellular or humoral defence reactions upon artificial bacterial challenge. Hence, the non-pathogenic *E. coli* caused a quick and complete arrest of pupal development accompanied by a premature darkening and decomposition of the whole pupal body, possibly as a consequence of a massive proliferation of *E. coli* bacteria in the haemolymph of infected pupae (Figures 6–8). Notably, these effects were even observed upon challenge of pupae with a low dose of *E. coli* (Figure 9). Honey bee pupae contain a high concentration of haemocytes [30] and also a fat body throughout their development [69], so that an absence of these immune-reactive tissues cannot be the reason for the lack of immune reactions.

Almost no data exist about the immune system of pupae, mostly because of their small size and limited availability in many insect orders – with one exception. Largely due to their giant size, silk moth larvae and pupae of the order Lepidoptera were among the first employed for the study of innate immune responses. The diapausing pupae of *Hyalophora cecropia* and of *Samia cynthia* were found to react with an inducible antibacterial activity in their haemolymph upon artificial infection with two Gram-negative bacteria [70], [71]. Later on, the first insect-specific antimicrobial peptides, named cecropins, were isolated from the haemolymph of *H. cecropia* pupae and their primary structure was elucidated [72]. The strong humoral immune response of silk moth pupae upon challenge with bacteria and the absence of any effective humoral or cellular defence reaction of honey bee pupae arises the question about the differences of their developmental life stages. After hatching, young silk moth caterpillars feed on leaves and at the end of the final instar stage begins to spin their cocoons which are attached along a twig and which consist of three layers protecting the pupae inside the cocoon from external damage. The development of honey bees contrasts with that of silk moths in many respects. Honey bee larvae do not care for their food themselves, but are nourished within their open brood cells with royal jelly produced and delivered by nurse bees. At this life stage, bee larvae are most susceptible to invasion by pathogenic bacteria by their daily replenished diet and hence are equipped with an adequate immune system (see above). In this context it should be mentioned that royal jelly contains various antimicrobial compounds among them the bee-specific AMP defensin 1 [66]–[68], [73] which minimize bacterial and fungal infections.

At the last larval or prepupal stage, bee larvae stretch out and begin to spin a cocoon around themselves. Subsequently, the brood cells are sealed with a wax lid by worker bees [1], [40]. This extraordinary seclusion of honey bee pupae might be one explanation for their loss of an active immune system. The prepupal stage is accomplished by defecation. As a consequence, bee pupae are usually free of intestinal microorganisms that larvae have ingested with their food [74]. This feature might further explain the absence of an active immune system in bee pupae which are neither externally nor internally threatened by microbial infections – until the recent introduction of the *Varroa* mite. The most deleterious effect of this new honey bee parasite is caused by the reproductive phase of *V. destructor* within sealed drone and worker brood cells. The mother mite creates a hole in the cuticle of the pupa, sucking out the haemolymph for feeding the offspring [75]. The bee pupa is damaged in a variety of ways that either leads to premature death of the pupa or results in a reduced life-span or an impairment of cognitive abilities of adult worker bees [75], [76]. Being a vector for various honey bee viruses, *Varroa* mite infestation of the brood might enhance the damaging effect on pupae and adults [41], [75]. Bacteria might as well be transmitted by mites within the brood cell from their former contacts with honey bee workers in the hive and hence, bacterial infections could in fact contribute to the overall damage of pupae because of the absence of defence reactions in the latter. In this context, it should be noted that bacterial colonies have frequently been found on the *Varroa* feeding sites of honey bee pupae [77].

The development of honey bee pupae is exceedingly fast due to stable high brood nest temperatures of 33–36°C [35]. The regulation of brood temperature is achieved by individual worker bees while pressing their thoraces onto capped brood cells [78]. Deviations of the temperature during pupal development will influence their behavioural performance as adults [79], [80]. It has been suggested that the activation and maintenance of immune reactions is costly and cannot be sustained together with other demanding activities [38]. Hence, it is conceivable to assume that bee pupae utilize all of their energy resources for morphogenetic-related metabolic processes.

Viral infections of insects apparently do not induce the same innate immune responses as bacteria, instead intrinsic defence mechanisms based on RNA interference play a major role in antiviral immunity in insects [81]. In accordance with these findings, we have recently demonstrated that infection of honey bee larvae and young adult worker bees with acute bee paralysis virus (ABPV) does not trigger humoral or cellular immune responses [29]. Honey bee pupae succumb to challenge with ABPV, albeit with some delay as compared to bacterial infections. Virus accumulation in bee pupae leads to a complete arrest of the pupal development 3–4 days post-infection (Figure 6). The reason for this phenomenon is not known but emphasizes the vulnerability of honey bee pupae.

Taken together, we have demonstrated in this report that honey bees rely on a great repertoire of innate immune reactions but that the activation of all components depends on their developmental life stages together with environmental hazards. Although not taking any risks, honey bees have apparently adapted a balance between the urgency to activate defence reactions and the feasibility to conserve energy.

## Materials and Methods

### Bacteria Strains, Viruses and Growth Conditions

The Gram-negative bacterium *Escherichia coli* (DSM 682) was obtained from the “Deutsche Sammlung von Mikroorganismen und Zellkulturen GmbH” (DSMZ, Braunschweig, Germany) and the Gram-positive bacterium *Micrococcus flavus* was a gift from Dr. U. Rdest (Institute of Microbiology, Biocentre, Würzburg, Germany). The *E. coli* strain was cultivated in nutrient broth (NB medium), whereas *M.flavus* was grown in lysogeny broth (LB medium) as described previously [23]. All ingredients were purchased from Becton Dickinson (Heidelberg, Germany). For infection experiments, *E. coli* bacteria were grown to an absorbance of A_550_ = 0.5 (∼3×10^8^ cells/ml). After centrifugation at 5200 rpm for 5 min (Eppendorf 5417R), cells were washed two times and resuspended in phosphate-buffered saline (PBS) at the desired concentration. A single highly purified suspension of Acute Bee Paralysis Virus (ABPV) prepared at the CVUA laboratory (Freiburg, Germany) served as a source for virus infection [29].

### Origin of Honey Bee Larvae, Pupae and Adults

During the summer seasons of 2008–2011, worker honey bee larvae, pupae and adult bees were obtained each year from two colonies with sister queens of *Apis mellifera carnica* maintained at the BEEstation (University of Würzburg). Drone combs and adult drones were obtained from additional colonies kept in the same apiary. Winter bees were collected in the winter seasons 2008/2009 and 2009/2010 from the outer area of the bee cluster from two different colonies each year (BEEstation, University of Würzburg).

### *In vitro* Rearing of Worker and Drone Larvae

Newly hatched larvae were collected from appropriate combs with a Swiss grafting tool and transferred to a 24-well tissue culture plate filled with a basic diet as previously described [23], [24]. The grafted larvae were maintained in an incubator at 35°C and 70% relative humidity. Each day, they were transferred to new tissue culture plates filled with fresh prewarmed diet.

### *In vitro* Maintenance of Adult Drones and Worker Bees

Newly emerged drones and worker bees were obtained from caged combs. These were placed in an incubator at 35°C and 65% relative humidity shortly before workers and drones, respectively, emerged from sealed cells. For each series of experiments, the collected bees were divided into groups of 10 individuals and kept in small cages in an incubator at 30°C (drones) and 35°C (workers). They were supplied with 50% (v/v) ApiInvert (Südzucker, Mannheim, Germany) *ad libitum* as recently described [24], [29].

### Artificial Infection of Larvae and Adults

Larvae were injected dorsally with 1 µl of 10^5^ viable *E. coli* bacteria. For injection we used disposable calibrated (1–5 µl) glass capillaries with fine tips [23], [24]. Adult drones and workers, respectively, were injected laterally between the second and third tergite of the abdomen with 1 µl of 10^5^ viable *E. coli* bacteria diluted in PBS [24], [29].

### Challenge of Bee Pupae with Bacteria and Virus

Drone and worker pupae were collected at the white-eye stage from sealed cells of appropriate combs. Lids of the cells were detached and the pupae were carefully removed from the cells with tweezers avoiding any kind of injury. They were transferred into empty 24-well tissue culture plates (Greiner, Frickenhausen, Germany, No. 662160) that were constantly kept on a warm pad to prevent chilling of the pupae. Subsequently, the tissue culture plates were kept in an incubator at 35°C and 70% relative humidity. For aseptic and septic injury, volumes of 1µl of buffer (PBS = phosphate-buffered saline), bacteria (10^2^ or 10^5^ viable *E. coli* bacteria) or virus (10^3^ ABPV particles) were injected laterally into the thorax without removing the pupae from the culture plates.

### Haemolymph Collection

At the indicated times after bacterial challenge, haemolymph was extracted from larvae, pupae and adult bees of all castes as described [23], [24] and transferred to reaction tubes containing mixtures of N-phenylthiourea (PTU) and aprotinin [23]. The samples were kept at −20°C until further analysis. The volume of haemolymph collected from single individuals depended on their age and life stage: worker larva (4 d) = 15–20 µl; worker pupa = 30–40 µl; worker adult (1 d) = 7–10 µl; winter bees = 8–10 µl; drone larva (6 d) = 30–35 µl; drone pupa = 40–50 µl; drone adult (1 d) = 10–15 µl.

### Inhibition-zone Assay

An aliquot (0.2 ml) of a fresh overnight culture of *Micrococcus flavus* was spread onto agar plates (Ø 9 cm) containing LB medium. As soon as the bacterial layer had been adsorbed, 1.5 µl of undiluted haemolymph samples of individual bees were applied as a droplet onto the plates with a pipette tip. As a positive control, lysozyme was employed at a concentration of 5 µg/µl [23], [24]. After 24 h of incubation in an incubator at 37°C, the clear zones of inhibition were documented by photography against a dark background. In principle, we obtained the same results when employing *E.coli* as indicator bacteria, however, the maximal size of clear zones remained smaller as compared to *M. flavus* so that we preferred the latter for the documentation of antimicrobial activities.

### Assay of Nodulation Reactions

Nodule formation was assessed in larvae, newly emerged adults and winter bees at selected times after injection of 10^5^ viable *E. coli* bacteria. Before analysis, adult bees were partially embedded in paraffin (Histosec, Merck, Darmstadt, Germany). The dorsal abdominal tergits of adult individuals were carefully removed and the melanised nodules were counted under a stereomicroscope (Olympus SZX7, Hamburg, Germany). Larvae were transferred onto a sheet of parafilm on top of a layer of paraffin and fixed dorsally with two needles. The ventral side was cut from the cranial to the caudal end (avoiding injury of the gut) and the larval skins were then turned over and fixed with four additional needles before counting the visible nodules in the haemocoel.

### Measurement of *E. coli* Survival in the Haemolymph of Challenged Bees

After injection of 10^5^ viable *E. coli* cells, the total haemolymph was extracted from challenged larvae, pupae and adults with fine-tipped glass capillaries at indicated times post-injection (p.i.) and 100 µl aliquots of the haemolymph (after appropriate pre-dilutions with pre-warmed sterile PBS) were spread onto agar plates (Ø 9 cm) containing NB medium. After 24 h of incubation at 37°C, the number of colony-forming units (CFUs) per agar plate were counted, multiplied with the dilution factor and adjusted to the total extracted amount of haemolymph in order to determine the actual number of surviving *E. coli* bacteria per bee. To determine the initial number of injected *E. coli* cells, an aliquot of the *E. coli* suspension was spread on agar plates after appropriate dilutions immediately before injection. All data are derived from at least two independent series of experiments.

### SDS Polyacrylamide Gel Electrophoresis

One-dimensional gel electrophoresis was carried out in vertical polyacrylamide slab gels containing 0.1% SDS with a 5% stacking gel on top of the separation gel [82]. Haemolymph samples (1–1.5 µl) were diluted with 2× concentrated sample buffer, heated for 3 min at 95°C and subjected to electrophoresis at constant voltage (120 V). For a better resolution of small proteins, the SDS PAGE system according to Schägger and von Jagow [83] was employed with some modifications [23]. After electrophoresis, gels were first fixed for 30 min in 0.85% o-phosphoric acid/20% methanol followed by colloidal Coomassie Blue G250 staining overnight in a solution of Roti®-Blue (Roth, Karlsruhe, Germany) and 20% methanol. Gels were destained in 25% methanol.

Two-dimensional gel electrophoresis was performed by combining isoelectrofocusing (IEF) on 18 cm IPG strips (GE Healthcare) pH range 3–10 NL for the first dimension and 15% polyacrylamide/0.1% SDS slab gels for the second dimension as recently described [24], [36].

## References

[1] Tautz J (2008) The buzz about bees. Berlin Heidelberg: Springer. 284 p.

[2] Stabentheiner A, Pressl H, Papst T, Hrassnigg N, Crailsheim K (2003) Endothermic heat production in honeybee winter clusters. J Exp Biol 206: 353–358. 12477904 10.1242/jeb.00082

[3] Fluri P (1993) Die Regulation der Lebensdauer bei Bienenarbeiterinnen. Schweiz Bienenztg 116: 624–629.

[4] Page RE Jr, Peng CY (2001) Aging and development in social insects with emphasis on the honey bee, *Apis mellifera* L. Exp Gerontol. 36: 695–711. 10.1016/s0531-5565(00)00236-9 11295509

[5] Wheeler DE, Buck N, Evans JD (2006) Expression of insulin pathway genes during the period of caste determination in the honey bee, *Apis mellifera* . Insect Mol Biol 15: 597–602. 17069635 10.1111/j.1365-2583.2006.00681.x PMC1761130

[6] Barchuk AR, Cristino AS, Kucharski R, Costa LF, Simoes ZL, et al (2007) Molecular determinants of caste differentiation in the highly eusocial honeybee *Apis mellifera* . BMC Dev Biol 7: 70. 17577409 10.1186/1471-213X-7-70 PMC1929063

[7] Patel A, Fondrk MK, Kaftanoglu O, Emore C, Hunt G, et al (2007) The making of a queen: TOR pathway is a key player in diphenic caste development. PLoS One 2: e509. 17551589 10.1371/journal.pone.0000509 PMC1876819

[8] Kucharski R, Maleszka J, Foret S, Maleszka R (2008) Nutritional control of reproductive status in honeybees via DNA methylation. Science 319: 1827–1830. 18339900 10.1126/science.1153069

[9] Kamakura M (2011) Royalactin induces queen differentiation in honeybees. Nature 473: 478–483. 21516106 10.1038/nature10093

[10] Jay C (1962) Colour changes in honeybee pupae Bee World. 43: 119–122.

[11] Rembold H, Kremer JP, Ulrich GM (1980) Characterization of postembryonic developmental stages of the female castes of the honey bee, *Apis mellifera* L.. Apidologie 11: 29–38.

[12] Michelette ER, Soares AEE (1993) Characterization of preimaginal developmental stages in africanized honey bee workers (*Apis mellifera* L). Apidologie 24: 431–440.

[13] Trenczek T (1998) Endogenous defense mechanisms of insects. Zoology 101: 298–315.

[14] Lemaitre B, Hoffmann J (2007) The host defense of Drosophila melanogaster. Annu Rev Immunol 25: 697–743. 17201680 10.1146/annurev.immunol.25.022106.141615

[15] Hultmark D (2003) Drosophila immunity: paths and patterns. Curr Opin Immunol 15: 12–19. 12495727 10.1016/s0952-7915(02)00005-5

[16] Honeybee Genome Sequencing Consortium (2006) Insights into social insects from the genome of the honeybee *Apis mellifera* . Nature 443: 931–949. 17073008 10.1038/nature05260 PMC2048586

[17] Evans JD, Aronstein K, Chen YP, Hetru C, Imler JL, et al (2006) Immune pathways and defence mechanisms in honey bees *Apis mellifera* . Insect Mol Biol 15: 645–656. 17069638 10.1111/j.1365-2583.2006.00682.x PMC1847501

[18] Casteels P, Ampe C, Jacobs F, Vaeck M, Tempst P (1989) Apidaecins: antibacterial peptides from honeybees. EMBO J 8: 2387–2391. 2676519 10.1002/j.1460-2075.1989.tb08368.x PMC401180

[19] Casteels P, Ampe C, Riviere L, Van Damme J, Elicone C, et al (1990) Isolation and characterization of abaecin, a major antibacterial response peptide in the honeybee (*Apis mellifera*). Eur J Biochem 187: 381–386. 2298215 10.1111/j.1432-1033.1990.tb15315.x

[20] Casteels P, Ampe C, Jacobs F, Tempst P (1993) Functional and chemical characterization of hymenoptaecin, an antibacterial polypeptide that is infection-inducible in the honeybee (*Apis mellifera*). J Biol Chem 268: 7044–7054. 8463238

[21] Casteels-Josson K, Zhang W, Capaci T, Casteels P, Tempst P (1994) Acute transcriptional response of the honeybee peptide-antibiotics gene repertoire and required post-translational conversion of the precursor structures. J Biol Chem 269: 28569–28575. 7961803

[22] Schlüns H, Crozier RH (2007) Relish regulates expression of antimicrobial peptide genes in the honeybee, Apis mellifera, shown by RNA interference. Insect Mol Biol 16: 753–759. 18093004 10.1111/j.1365-2583.2007.00768.x

[23] Randolt K, Gimple O, Geissendörfer J, Reinders J, Prusko C, et al (2008) Immune-related proteins induced in the hemolymph after aseptic and septic injury differ in honey bee worker larvae and adults. Arch Insect Biochem Physiol 69: 155–167. 18979500 10.1002/arch.20269

[24] Gätschenberger H, Gimple O, Tautz J, Beier H (2012) Honey bee drones maintain humoral immune competence throughout all life stages in the absence of vitellogenin production. J Exp Biol 215: 1313–1322. 22442369 10.1242/jeb.065276

[25] Strand MR (2008) The insect cellular immune response. Insect Science 15: 1–14.

[26] Aumeier P, Schmidt F, Trenczek T, Boecking O, Wittmann D (2001) Characterization of honey bee hemocytes. Apidologie 32: 491–492.

[27] Szymaś B, Jędruszuk A (2003) The influence of different diets on haemocytes of adult worker honey bees, *Apis mellifera* . Apidologie 34: 97–102.

[28] Bedick JC, Tunaz H, Nor Aliza AR, Putman SM, Ellis MD, et al (2001) Eicosanoids act in nodulation reactions to bacterial infections in newly emerged adult honey bees, *Apis mellifera*, but not in older foragers. Comp Biochem Physiol Part C 130: 107–117. 10.1016/s1532-0456(01)00226-5 11544147

[29] Azzami K, Ritter W, Tautz J, Beier H (2012) Infection of honey bees with acute bee paralysis virus does not trigger humoral or cellular immune responses. Arch Virol 157: 689–702. 22258854 10.1007/s00705-012-1223-0 PMC3314816

[30] Wilson-Rich N, Dres ST, Starks PT (2008) The ontogeny of immunity: Development of innate immune strength in the honey bee (*Apis mellifera*). J Insect Physiol 54: 1392–1399. 18761014 10.1016/j.jinsphys.2008.07.016

[31] Schmid MR, Brockmann A, Pirk CWW, Stanley DW, Tautz J (2008) Adult honeybees (*Apis mellifera* L.) abandon hemocytic, but not phenoloxidase-based immunity. J Insect Physiol 54: 439–444. 18164310 10.1016/j.jinsphys.2007.11.002

[32] Laughton AM, Boots M, Siva-Jothy MT (2011) The ontogeny of immunity in the honey bee, *Apis mellifera* L. following an immune challenge. J Insect Physiol 57: 1023–1032. 21570403 10.1016/j.jinsphys.2011.04.020

[33] Siede R, Meixner MD, Büchler R (2012) Comparison of transcriptional changes of immune genes to experimental challenge in the honey bee (*Apis mellifera*). J Apicult Res 51: 320–328.

[34] Fedorova AA, Azzami K, Ryabchikova EI, Spitsyna YE, Silnikov VN, et al (2011) Inactivation of a non-enveloped RNA virus by artificial ribonucleases: Honey bees and Acute bee paralysis virus as a new experimental model for in vivo antiviral activity assessment. Antiviral Res 91: 267–277. 21722669 10.1016/j.antiviral.2011.06.011

[35] Seeley TD (1985) Honeybee Ecology, A study of adaptation in social life. Princeton: Princeton Univ. Press. 201 p.

[36] Albert S, Gätschenberger H, Azzami K, Gimple O, Grimmer G, et al (2011) Evidence of a novel immune responsive protein in the Hymenoptera. Insect Biochem Mol Biol 41: 968–981. 22001069 10.1016/j.ibmb.2011.09.006

[37] Horohov DW, Dunn PE (1983 ) Phagocytosis and nodule formation by hemocytes of *Manduca sexta* larvae following injection of *Pseudomonas aeruginosa* . J Invertebr Pathol 41: 203–213.

[38] Moret Y, Schmid-Hempel P (2000) Survival for immunity: the price of immune system activation for bumblebee workers. Science 290: 1166–1168. 11073456 10.1126/science.290.5494.1166

[39] Azzami K, Gätschenberger H, Gimple O, Beier H, Tautz J (2009) Do winter bees have an immune system that is weaker than that of summer bees? Apidologie 40: 660.

[40] Winston ML (1987) The Biology of the Honey Bee. Cambridge: Harvard University Press. 294 p.

[41] Genersch E, Aubert M (2010) Emerging and re-emerging viruses of the honey bee (*Apis mellifera* L.). Vet Res 41: 54. 20423694 10.1051/vetres/2010027 PMC2883145

[42] Cox-Foster DL, Conlan S, Holmes EC, Palacios G, Evans JD, et al (2007) A metagenomic survey of microbes in honey bee colony collapse disorder. Science 318: 283–287. 17823314 10.1126/science.1146498

[43] Dainat B, Evans JD, Chen YP, Gauthier L, Neumann P (2012) Predictive markers of honey bee colony collapse. PLoS ONE 7: e32151. 22384162 10.1371/journal.pone.0032151 PMC3285648

[44] Gregory PG, Evans JD, Rinderer T, de Guzman L (2005) Conditional immune-gene suppression of honeybees parasitized by *Varroa mites* . J Insect Sci 5: 7. 16299597 10.1093/jis/5.1.7 PMC1283888

[45] Yang X, Cox-Foster DL (2005) Impact of an ectoparasite on the immunity and pathology of an invertebrate: evidence for host immunosuppression and viral amplification. Proc Natl Acad Sci USA 102: 7470–7475. 15897457 10.1073/pnas.0501860102 PMC1140434

[46] Moret Y, Schmid-Hempel P (2001) Immune defence in bumble-bee offspring. Nature 414: 506. 10.1038/35107138 11734840

[47] Doums C, Moret Y, Benelli E, Schmid-Hempel P (2002) Senescence of immune defence in *Bombus* workers. Ecol Entomol 27: 138–144.

[48] Kraus FB, Neumann P, Scharpenberg H, Van Praagh J, Moritz RFA (2003) Male fitness of honeybee colonies (*Apis mellifera* L.). J Evol Biol 16: 914–920. 14635906 10.1046/j.1420-9101.2003.00593.x

[49] Hrassnigg N, Crailsheim K (2005) Differences in drone and worker physiology in honeybees (*Apis mellifera*). Apidologie 36: 255–277.

[50] Dunn PE, Drake DR (1983) Fate of bacteria injected into naive and immunized larvae of the tobacco hornworm *Manduca sexta* . J Invert Pathol 41: 77–85.

[51] Korner P, Schmid-Hempel P (2004) In vivo dynamics of an immune response in the bumble bee *Bombus terrestris* . J Invertebr Pathol 87: 59–66. 15491600 10.1016/j.jip.2004.07.004

[52] Haine ER, Pollitt LC, Moret Y, Siva-Jothy MT, Rolff J (2008) Temporal patterns in immune responses to a range of microbial insults (*Tenebrio molitor*). J Insect Physiol 54: 1090–1097. 18513740 10.1016/j.jinsphys.2008.04.013

[53] Haine ER, Moret Y, Siva-Jothy MT, Rolff J (2008) Antimicrobial defense and persistent infection in insects. Science 322: 1257–1259. 19023083 10.1126/science.1165265

[54] Münch D, Amdam GV, Wolschin F (2008) Ageing in a eusocial insect: molecular and physiological characteristics of life span plasticity in the honey bee. Funct Ecol 22: 407–421. 18728759 10.1111/j.1365-2435.2008.01419.x PMC2525450

[55] Seehuus SC, Norberg K, Gimsa U, Krekling T, Amdam GV (2006) Reproductive protein protects functionally sterile honey bee workers from oxidative stress. Proc Natl Acad Sci USA 103: 962–967. 16418279 10.1073/pnas.0502681103 PMC1347965

[56] Fluri P, Wille H, Gerig L, Lüscher M (1977) Juvenile hormone, vitellogenin and hemocyte composition in winter worker honeybees (*Apis mellifera*). Experientia 33: 1240–1241.

[57] Stanley DW, Miller JS (2006) Eicosanoid actions in insect cellular immune functions. Entomol Exp Appl 119: 1–13.

[58] Stanley-Samuelson DW, Jensen E, Nickerson KW, Tiebel K, Ogg CL, et al (1991) Insect immune response to bacterial infection is mediated by eicosanoids. Proc Natl Acad Sci USA 88: 1064–1068. 1899480 10.1073/pnas.88.3.1064 PMC50955

[59] Stanley D (2011) Eicosanoids: progress towards manipulating insect immunity. J Appl Entomol 135: 534–545.

[60] Cerenius L, Lee BL, Söderhäll K (2008) The proPO-system: pros and cons for its role in invertebrate immunity. Trends Immunol 29: 263–271. 18457993 10.1016/j.it.2008.02.009

[61] Behrends A, Scheiner R (2010) Learning at old age: a study on winter bees. Front Behav Neurosci 4: 15. 20428511 10.3389/fnbeh.2010.00015 PMC2859878

[62] Yue D, Nordhoff M, Wieler LH, Genersch E (2008) Fluorescence in situ hybridization (FISH) analysis of the interactions between honeybee larvae and *Paenibacillus larvae*, the causative agent of American foulbrood of honeybees (*Apis mellifera*). Environ Microbiol 10: 1612–1620. 18331334 10.1111/j.1462-2920.2008.01579.x

[63] Bamrick JF, Rothenbuhler WC (1961) Resistance to American foulbroud in honey bees, IV. The relationship between larval age at inoculation and mortality in a resistant and a susceptible line. J Insect Pathol 3: 381–390.

[64] Brødsgaard CJ, Ritter W, Hansen H (1998) Response of in vitro reared honey bee larvae to various doses of *Paenibacillus larvae larvae* spores. Apidologie 29: 569–578.

[65] Evans JD (2004) Transcriptional immune responses by honey bee larvae during invasion by the bacterial pathogen, *Paenibacillus larvae* . Journal of Invertebrate Pathology 85: 105–111. 15050840 10.1016/j.jip.2004.02.004

[66] Fujiwara S, Imai J, Fujiwara M, Yaeshima T, Kawashima T, et al (1990) A potent antibacterial protein in royal jelly. Purification and determination of the primary structure of royalisin. J Biol Chem 265: 11333–11337. 2358464

[67] Klaudiny J, Albert S, Bachanova K, Kopernicky J, Simuth J (2005) Two structurally different defensin genes, one of them encoding a novel defensin isoform, are expressed in honeybee *Apis mellifera* . Insect Biochem Mol Biol 35: 11–22. 15607651 10.1016/j.ibmb.2004.09.007

[68] Bíliková K, Wu G, Šimúth J (2001) Isolation of a peptide fraction from honeybee royal jelly as a potential antifoulbrood factor. Apidologie 32: 275–283.

[69] Ivanova E, Staikova T (2007) Stage specifity in the expression of proteins of honey bee fat body (*Apis mellifera* L.) in the course of ontogenesis. J Cell Mol Biol 6: 129–135.

[70] Boman HG, Nilsson-Faye I, Paul K, Rasmuson T Jr (1974) Insect immunity. I. Characteristics of an inducible cell-free antibacterial reaction in hemolymph of *Samia cynthia* pupae. Infect Immun 10: 136–145. 4210336 10.1128/iai.10.1.136-145.1974 PMC414969

[71] Faye I, Pye A, Rasmuson T, Boman HG, Boman IA (1975) Insect immunity. II. Simultaneous induction of antibacterial activity and selective synthesis of some hemolymph proteins in diapausing pupae of *Hyalophora cecropia* and *Samia cynthia* . Infect Immun 12: 1426–1438. 812827 10.1128/iai.12.6.1426-1438.1975 PMC415452

[72] Steiner H, Hultmark D, Engström A, Bennich H, Boman HG (1981) Sequence and specificity of two antibacterial proteins involved in insect immunity Nature. 292: 246–248. 10.1038/292246a0 7019715

[73] Fujita T, Kozuka-Hata H, Ao-Kondo H, Kunieda T, Oyama M, et al (2013) Proteomic analysis of the royal jelly and characterization of the functions of its derivation glands in the honeybee. J Proteome Res 12: 404–411. 23157659 10.1021/pr300700e

[74] Gilliam M, Prest DB (1987) Microbiology of feces of the larval honey bee, *Apis mellifera* . J Invertebr Pathol 49: 70–75.

[75] Rosenkranz P, Aumeier P, Ziegelmann B (2010) Biology and control of *Varroa destructor* . J Invertebr Pathol 103: S96–S119. 19909970 10.1016/j.jip.2009.07.016

[76] Kralj J, Brockmann A, Fuchs S, Tautz J (2007) The parasitic mite *Varroa destructor* affects non-associative learning in honey bee foragers, *Apis mellifera* L. J Comp Physiol A Neuroethol Sens Neural Behav Physiol. 193: 363–370. 10.1007/s00359-006-0192-8 17123087

[77] Kanbar G, Engels W (2003) Ultrastructure and bacterial infection of wounds in honey bee (*Apis mellifera*) pupae punctured by Varroa mites. Parasitol Res 90: 349–354. 12684884 10.1007/s00436-003-0827-4

[78] Bujok B, Kleinhenz M, Fuchs S, Tautz J (2002) Hot spots in the bee hive. Naturwissenschaften 89: 299–301. 12216858 10.1007/s00114-002-0338-7

[79] Tautz J, Maier S, Groh C, Rössler W, Brockmann A (2003) Behavioral performance in adult honey bees is influenced by the temperature experienced during their pupal development. Proc Natl Acad Sci USA 100: 7343–7347. 12764227 10.1073/pnas.1232346100 PMC165877

[80] Becher MA, Scharpenberg H, Moritz RFA (2009) Pupal developmental temperature and behavioral specialization of honeybee workers (*Apis mellifera* L.). J Comp Physiol A 195: 673–679. 10.1007/s00359-009-0442-7 19390855

[81] Huszar T, Imler J-L (2008) *Drosophila* viruses and the study of antiviral host-defense. Adv Virus Res 72: 227–265. 19081493 10.1016/S0065-3527(08)00406-5

[82] Laemmli UK (1970) Cleavage of structural proteins during the assembly of the head of bacteriophage T4. Nature 227: 680–685. 5432063 10.1038/227680a0

[83] Schägger H, von Jagow G (1987) Tricine-sodium dodecyl sulphate-polyacrylamide gel electrophoresis for the separation of proteins in the range from 1 to 100 kDa. Anal Biochem 166: 368–379. 2449095 10.1016/0003-2697(87)90587-2

